# Development and Validation of a High-Throughput Quantification Method of Crown Procyanidins in Different Wines by UHPLC-Q-TOF

**DOI:** 10.3390/mps8010007

**Published:** 2025-01-11

**Authors:** Manon Ferreira, Pierre-Louis Teissedre, Michaël Jourdes

**Affiliations:** Univ. Bordeaux, INRAE, Bordeaux INP, Bordeaux Sciences Agro, UMR 1366, OENO, ISVV, F-33882 Villenave d’Ornon, France; manon.ferreira.1@u-bordeaux.fr (M.F.); pierre-louis.teissedre@u-bordeaux.fr (P.-L.T.)

**Keywords:** crown procyanidins, UHPLC-Q-TOF, quantification, validation, wine

## Abstract

Procyanidins are widely distributed in plant-derived foods, and consist of flavanol oligomers and polymers. Recently, the crown procyanidin sub-family, characterised by a unique macrocyclic structure, has been identified in grapes and wine. This study reports the development and validation of a rapid and quantitative analytical method measuring crown procyanidin concentration in red and white wines using ultra-high-performance liquid chromatography (UHPLC) coupled with a Q-TOF mass spectrometer. Validation followed international standards, demonstrating high sensitivity (LOQ = 0.033 mg/L), accuracy (recovery = 88.21% to 107.64%), repeatability (RSD = 1.99% to 11.03%), and intermediate reproducibility (RSD = 2.51% to 19.05%). Minimal matrix effects were observed, ensuring reliable and precise quantification across both wine types. The applicability of the method was confirmed through the successful analysis of wine samples, leading to the first quantification of crown procyanidins in white wine. Concentrations ranged from 0.81 mg/L to 15.88 mg/L in the different analysed wines. This validated method provides a valuable tool for the study of crown procyanidin profiles in various wine matrices and establishes a foundation for future research into the role of crown procyanidins in wines and other food and beverage matrices where these compounds may be present.

## 1. Introduction

Condensed tannins (also called proanthocyanidins) are widely distributed in plant-derived foods and beverages, like grapes, red wine, nuts, tea, apples, and chocolate [1]. These molecules are extensively studied for their ability to interact with other polyphenols or compounds. In fact, in wine, they contribute to various organoleptic properties, such as astringency and bitterness [2,3], and they are also involved in the color stability and evolution of red wines [4,5]. During the winemaking process, these condensed tannins are extracted from the skins and seeds of the grape, and their concentrations in red wine are influenced by the grape variety, as well as by the winemaking process [6,7]. Condensed tannins are oligomers and polymers of flavan-3-ol units linked through C4-C8 or C4-C6 interflavanol linkages. In grapes, the five flavanol monomers involved in the condensed tannins differ in the degree of hydroxylation on their B rings, as well as the configuration of their asymmetric carbon in C3 and the presence of a galloyl group link at this position [8,9,10,11]. The main monomeric flavan-3-ols in grapes include (+)-catechin and (-)-epicatechin, which have a catechol-type B ring, as well as (+)-gallocatechin and (-)-epigallocatechin, with additional hydroxylation on their B rings. Finally, (-)-epicatechin-3-*O*-gallate results in the esterification of gallic acid at position C3 of the epicatechin.

**Figure 1:**
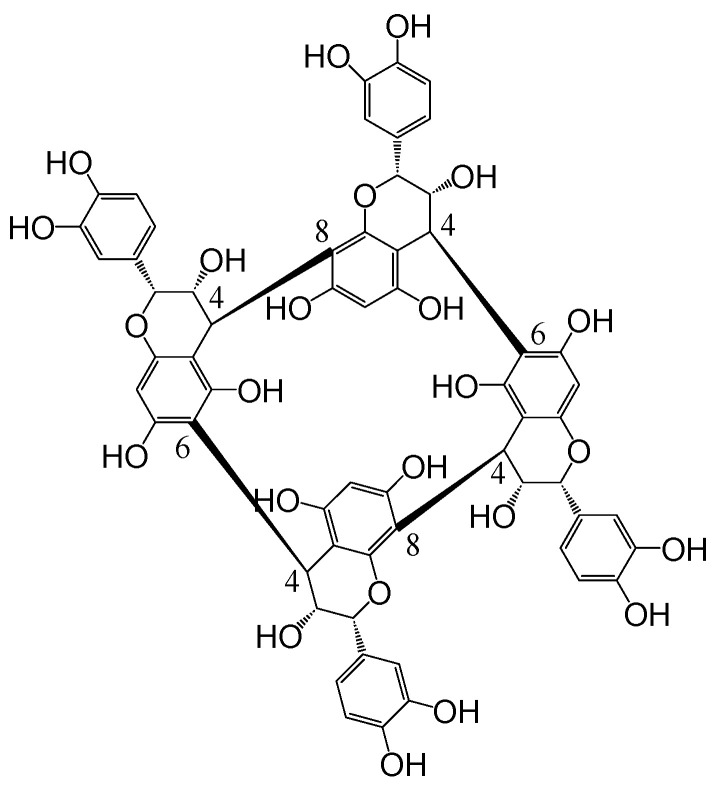
Structure of crown procyanidin tetramer.

In 2015, a new sub-family of condensed tannins with an unusual structure was reported, named crown procyanidins. The structure of the crown procyanidin tetramer (Figure 1) has been characterised by NMR, which revealed a macrocyclic structure composed of four (-)-epicatechin monomers [12]. To date, in dry red wine, 11 other crown tannins have been detected by MS-MS, including tetramers, pentamers, and hexamers [13,14,15]. Their abundance in grape skin depends on the grape variety; to date, around fifty different grape varieties (white and red varieties) have been analysed and all of them exhibit these tannins [14,16]. In addition, other properties show that these crown procyanidins are very different compared to regular condensed tannins. Indeed, in contrast with regular condensed tannins, crown procyanidins are extracted at the beginning of maceration and alcoholic fermentation, like anthocyanins, which indicates that crown tannins are more soluble in water than non-cyclic condensed tannins. Moreover, crown procyanidins appear to be more resistant to oxidation in wine than regular condensed tannins [17]. However, some of these results are expressed as the relative MS abundance, without a calibration curve or the use of validated methods using a pure crown procyanidin standard, or they are obtained from a single external calibration curve.

The separation and quantification of oligomeric condensed tannins is generally a complex challenge, especially due the large diversity in the structures of these compounds. Among the various LC platforms, ultra-performance liquid chromatography (UPLC) holds advantages over HPLC due to its increased resolution, higher sensitivity, excellent peak shapes, and enhanced reproducibility [18,19,20]. In recent years, high-resolution mass spectrometry (HRMS), including Q-TOF-MS, has gained popularity because of its ability to provide mass information with greater accuracy and precision, as well as some structural information [21,22,23]. Moreover, Q-TOF mass spectrometry exhibits a wider linear or dynamic range than other MS techniques, allowing accurate detection across a broad concentration range. This enhances its utility for diverse and complex samples.

In line with our knowledge regarding the identification and structural characterisation of crown procyanidins, the objective of this study was to develop and validate a method for the rapid and reliable quantification of crown tannins for various oenological matrices (i.e., in red and white wines) by UPLC-Q-TOF-MS.

## 2. Materials and Methods

### 2.1. Chemicals

Ultrapure water (Milli-Q purification system, Millipore, Guyancourt, France) was used for all solutions and chromatographic separation. Methanol (MeOH, 100%, VWR, Rosny-sous-Bois, France), acetone (100%, VWR), and formic acid (purity ≥ 99.3%, Fisher Scientific, Illkirch, France,) were used for TSK cartridge fractionation and semi-preparative HPLC. The methanol (Optimal^®^ LC/MS) and formic acid (Optimal^®^ LC/MS) that were used for high-resolution mass spectrometry analysis were obtained from Fisher Scientific (Geel, Belgium).

### 2.2. Wines

The experimental assays for the validation of the liquid chromatographic method were carried out using commercially available red and white wines (bag in box). The different red and white wines quantified were obtained from the microvinification performed at the Institute of Vine and Wine Sciences at the University of Bordeaux, from grapes collected at technical maturity.

### 2.3. Crown Tetramer Procyanidin Purification

#### 2.3.1. TSK HW-50F Fractionation

First, 50 mL of Clairet wine was evaporated to remove the ethanol present and then loaded onto a Toyopearl TSK HW-50F gel column (d: 5cm, h: 17cm) (Tosoh, Tokyo, Japan). The column was conditioned with MeOH/H_2_O (80/20, *v*/*v*) with 0.1% formic acid. Successive elution was achieved as follows: (1) 500 mL MeOH/water (80/20, *v*/*v*), (2) 250 mL acetone/water (30/70, *v*/*v*), and (3) 500 mL acetone/water (85/15, *v*/*v*). All solvents used were acidified with 0.1% formic acid. The fraction obtained with acetone/water (85/15, *v*/*v*) was collected, evaporated to dryness, and then resolubilised in 1 mL of milliQ water acidified with 0.1% formic acid for subsequent purification by semi-preparative HPLC-UV.

#### 2.3.2. Semi-Preparative HPLC-UV Purification

A semi-preparative HPLC-UV system was used to obtain the pure crown procyanidin tetramer. The system was composed of two pumps (Varian ProStar model 210, Les Ulis, France), an external manual sample injector with a 2 mL injection loop, and a diode array detector (Varian ProStar Model 325, Les Ulis, France). The whole system was controlled by the Varian Star software (6.41 Chromatography Workstation). The manual injection volumes varied from 500 to 1500 µL. The semi-preparative column was a Nucleodur C18 HTec column (250 × 21 mm, 5 µm) from Macherey-Nagel. The UV detection was set at 280 nm. The mobile phases were solvent A (milliQ water acidified to 0.1% with formic acid) and solvent B (methanol acidified to 0.1% with formic acid), with a flow rate of 10 mL/min. A pool of TSK fractions was subsequently injected to purify the crown procyanidin tetramer with the following solvent B elution gradient: 0 min, 6%; 40 min, 12%; 44 min, 99%; 50 min, 99%; 51 min, 6%; 54 min, 6%. The compound was collected in 21 to 22 min, evaporated to dryness, and then resolubilised in milliQ water, frozen, and lyophilised in order to obtain the crown procyanidin tetramer as a pure white powder.

Two litres of Clairet wine (Bordeaux vintage 2023) was purified using this two-step procedure, leading to 1.1 mg of pure crown procyanidin tetramer as a white powder.

### 2.4. Sample Preparation and UHPLC-MS Analysis for Crown Procyanidin Quantification

Prior to UHPLC-Q-TOF injection, red wines were diluted 20 times and white wines were diluted 10 times in water and were filtered through a 0.45 µm filter. The UHPLC-Q-TOF system used was an Agilent 1290 Infinity equipped with a binary pump (1290 Infinity), a thermostated column compartment (1290 Infinity), an autosampler module (1290 Infinity), and a diode array detector (1290 Infinity), which was coupled to an ESI-Q-TOF-MS (Agilent 6530 Accurate Mass, Les Ulis, France). Chromatographic separation was carried out on an Eclipse Plus C18 column (2.1 × 100 mm, 1.8 μm). The solvents used were water with 0.1% formic acid for solvent A and methanol with 0.1% formic acid for solvent B, at a flow rate of 0.3 mL/min. The gradient of solvent B for crown procyanidin analysis was as follows: 0 min, 4%; 0.5 min 6%, 29%; 4 min, 95%; 5 min, 95%; 10 min. The column was equilibrated for 3 min using the starting condition. The ESI conditions were as follows: gas temperature and flow rate were set at 300 °C and 9 L/min, respectively; sheath gas temperature and flow rate were set at 350 °C and 11 L/min, respectively; capillary voltage was set at 4000 V. The fragmentor was always set at 200 V. The sensitivity of the instrument was controlled with check tune calibration. The crown procyanidin tetramer (*m*/*z* 1153.2614), the crown procyanidin mono-gallo-tetramer (*m*/*z* 1169.2563), and the crown procyanidin pentamer (*m*/*z* 1441.3248) were detected via UHPLC-Q-TOF. Regarding the quantification methods, an external calibration curve using the purified crown procyanidin tetramer (0–1 mg/L) was injected for each wine’s quantification. The crown procyanidin mono-gallo-tetramer and pentamer were expressed as mg/L eq. crown procyanidin tetramer. Data processing was carried out with the MassHunter qualitative analysis software (version B.05).

### 2.5. UHPLC-Q-TOF Method Validation

The validation of the analytical method was carried out by assessing the following parameters: the matrix effect, linearity, accuracy, repeatability, intermediate reproducibility, limits of detection (LOD), and limits of quantification (LOQ).

The matrix effect was measured by comparing the signal obtained in water and in wine samples spiked with the crown procyanidin tetramer at the same level of concentration. Red wine was diluted 20 times and white wine was diluted 10 times. The two wines were then spiked with the crown procyanidin tetramer at 0 and 0.1 mg/L in triplicate, and the signal corresponding to the enrichment was compared with water doped at 0.1 mg/L. At that time, the matrix effect was evaluated by calculating the Z-score parameter. If the Z-score was inferior to 2, it could be concluded that there was no matrix effect between the water and wine samples [24].

Linearity was calculated by spiking water at nine crown procyanidin tetramer concentrations (0–1 mg/L) in triplicate and by plotting the area against the respective crown procyanidin tetramer concentration. The range was chosen according to the diluted wine matrices. Subsequently, linearity was evaluated by a lack of fit test [25].

The limit of detection (LOD) and the limit of quantification (LOQ) were defined by the standard deviation of the response and the slope of the calibration curve. The LOD and LOQ corresponded to 3.3σ/slope and 10σ/slope, respectively. In order to confirm that the analytes could be accurately detected and quantified, the values were then verified by visually assessing the chromatograms from the analysis of the samples at the estimated LOD and LOQ.

The accuracy, repeatability, and intermediate reproducibility were measured by spiking real samples at three concentrations. The results were defined by the recovery (concentrations calculated from calibration compared with theoretical concentrations, expressed in %) and mean RSD (%). For wine, samples of diluted red and white wines were spiked with three levels of crown procyanidin tetramer in triplicate. For the repeatability and accuracy, the levels were 0.1, 0.25, and 0.5 mg/L. For the reproducibility, the levels were 0.05, 0.1, and 0.5 mg/L.

### 2.6. UHPLC-MS/MS Analysis for Crown Procyanidin Identification

Prior to the quantitative analysis, we performed UHPLC-MS/MS analyses to assess the crown proanthocyanidins. The UHPLC parameters were identical to those defined in Section 2.4. For the mass parameters, the ESI conditions were identical to those used for the MS mode. Here, we used the targeted MS/MS mode (seg); the parameters were identical to those used for mass MS, with the exception of the fragmentation parameters. We targeted the 3 crown tannins mentioned above (i.e., *m*/*z* 1153.2614, *m*/*z* 1169.2563, and *m*/*z* 1441.3248). Collision energy of 30 eV was applied for the first two molecules and 35 eV for the last. Data processing for these analyses was also carried out using the MassHunter qualitative analysis software (version B.05).

### 2.7. Statistical Analyses

The statistical analyses were carried out using the R software with version 4.4.0. In order to highlight significant differences between the total crown procyanidin concentrations in the different wines, a one-way analysis of variance (ANOVA) was performed, taking the wine type as a factor. To differentiate the means, Tukey’s post hoc test was applied. Differences with a *p*-value < 0.05 were considered statistically significant.

## 3. Results

### 3.1. HPLC Method Development

The developed methodology for the quantification of crown procyanidins in wine samples was based on a previously reported method [13]. Regarding the UPLC separation, all key parameters (i.e., stationary phase, gradient, solvent, flow rate, column temperature, and injection volume) were optimised to achieve the best separation of the studied crown procyanidins in the shortest time. The run time was optimised to 10 min, with a post-run time of 3 min to equilibrate the column. Regarding the detection by the Q-TOF mass spectrometer, the main parameters, such as the sheath gas temperature and flow rate, capillary voltage, and fragmentor, were selected to obtain the highest signal with the lowest noise during sample analysis.

### 3.2. Validation of Quantification Method by UHPLC-Q-TOF

Validation was performed according to the recommendations of the International Organisation of Vine and Wine (OIV) (OIV-MA-AS1-12). The pure crown procyanidin tetramer, obtained through two-step purification from Clairet wine, was used as a standard in the validation of this quantification method.

#### 3.2.1. Matrix Effect

In the validation procedure, the matrix effect was the first parameter that needed to be measured. Indeed, this parameter allowed us to evaluate the specificity degree of the method. It also enabled us to determine whether linearity could be established under standard conditions (using water) or in a real matrix (i.e., wine). In practice, water and real samples were spiked at two concentrations (C0 = no spiking and C1 = 0.1 mg/L after dilution). As shown in the table, the Z-scores were lower than two, indicating that there was no significant matrix effect for the two studied matrices (i.e., red wine and white wine) (Table 1). As a result, linearity was achieved under standard conditions, providing clear practical advantages for the next step of the method’s validation, as well as for the overall analytical method. This will allow analyses of a variety of samples without requiring complex sample preparation, as with a spiking method.

#### 3.2.2. Linearity

After the matrix effect study, the calibration curve was plotted. In practice, the concentrations were plotted against the corresponding area obtained. To check the linearity, the lack of fit test was applied [25]. The linearity of the method was assessed by evaluating the quality of the fit through back-calculated to nominal concentrations and employing the F-test to confirm the method’s linearity. The calculated F value was lower than the critical F value of the Fisher–Snedecor table for α = 0.05 (5%). The linear model was deemed to be suitable for our quantified compound as the R^2^ value was above 0.9919 (see supporting information). Consequently, in the subsequent validation experiments, linear models were used to calculate the limit of detection (LOD) and limit of quantification (LOQ) and to evaluate the accuracy.

#### 3.2.3. Limit of Detection (LOD) and Limit of Quantification (LOQ)

The limit of detection (LOD) and limit of quantification (LOQ) were established using the standard deviation of the response and the slope of the calibration curve. The two formulas 3.3σ/slope and 10σ/slope were employed to calculate the LOD and LOQ, resulting in values of 0.011 mg/L and 0.033 mg/L, respectively.

#### 3.2.4. Accuracy

The accuracy was determined by adding known amounts of the crown procyanidin tetramer to two different wines (i.e., red and white wine) at three different concentration levels (i.e., 0.1, 0.25, and 0.5 mg/L). Unspiked control samples were also analysed to distinguish the initial levels of the compound from the added amounts. The accuracy was evaluated by comparing the detected concentrations with the theoretical values of the spiked samples. The method demonstrated accuracy ranging from 88.21% to 107.64% for the compound under the test conditions, which aligns with the OIV standards, where an accuracy range between 80% and 120% is deemed acceptable.

#### 3.2.5. Fidelity

The fidelity parameter involves the assessment of the repeatability and intermediate reproducibility. The repeatability was evaluated by spiking two types of wine matrices (i.e., red and white wine) at three concentration levels (i.e., 0.1, 0.25, 0.5 mg/L) in triplicate. The relative standard deviation (RSD) was calculated for each concentration level and typically reported as a single value for homoscedastic series. The resulting RSD values ranged from 1.99% to 11.03%.

The intermediate reproducibility was assessed over five days by analysing the same spiked wine samples (diluted red and white wines) at three different concentrations (0.05, 0.1, 0.5 mg/L), with the RSD of the measured concentrations calculated under consistent conditions (same sample, analyst, and equipment). Similar to the repeatability, the RSD values for the intermediate reproducibility were acceptable, ranging from 2.51% to 19.05%. The fidelity parameters were validated and demonstrated to be highly satisfactory for the new quantification method, as the OIV guidelines recommend repeatability and intermediate reproducibility values below 20%.

### 3.3. Identification of Crown Procyanidins by UHPLC-Q-TOF-MS/MS

The detected crown procyanidins were first fragmented in order to confirm that they belonged to the crown tannin sub-family prior to their quantification in the wine samples. The fragments of the various crown tannins are reported in Table 2.

The MS-MS spectrum obtained for the crown procyanidin tetramer [C_60_H_49_O_25_] (*m*/*z* = 1169.2588) revealed classical fragmentation patterns specific to a flavanol unit [26,27,28]. Indeed, it revealed fragments resulting from Retro-Diels–Alder (RDA) fission with a loss of −152.0473 Da (-C_8_H_8_O_3_, leading to ion fragments at *m*/*z* 1017.2048, at *m*/*z* 729.1456, at *m*/*z* 713.1552, at *m*/*z* 441.0851, and at *m*/*z* 425.0873) or −168.0423 Da (-C_8_H_8_O_4_, fragments at *m*/*z* 713.1552 and at *m*/*z* 425.0873) when the RDA fission was located on the gallo-flavanol unit. Fragments from hydrogen rearrangement fission (HRF) with a loss of −126.0317 Da (-C_6_H_6_O_3_, producing ions fragment at *m*/*z* 755.1612, at *m*/*z* 739.1755, at *m*/*z* 467.0955, and at *m*/*z* 451.1021) were also observed, as well as similar fragments with a loss of −142.0266 Da (-C_6_H_6_O_4_, fragment at *m*/*z* 739.1755 and at *m*/*z* 451.1021) resulting from an HRF fission on the epigallocatechin or gallocatechin unit. Moreover, multiple ions with a loss of 288.0634 Da or 304.0583 Da (C_15_H_12_O_6_ or C_15_H_12_O_7_) were observed at *m*/*z* 1169.2551, *m*/*z* 881.1918, *m*/*z* 865.1924, *m*/*z* 593.1310, *m*/*z* 577.1309, *m*/*z* 305.0633, and *m*/*z* 289.0702, resulting from successive QM fissions from this crown mono-gallo-proanthocyanidin tetramer (*m*/*z* 1169.2588). As with the classical crown procyanidin tetramer, the crown mono-gallo-proanthocyanidin tetramer, once ionised, can undergo the rearrangement of the terminal unit, which prevents the loss of −290.0790 Da. Therefore, only losses of 288.0634 Da and 304.0583 Da (plus -OH) were observed during crown tetramer fragmentation. Finally, fragments corresponding to water loss (−18.0106 Da) were also observed (*m*/*z* 1151.2519; *m*/*z* 999.1925; 981.1836; *m*/*z* 847.1735; *m*/*z* 695.1406; *m*/*z* 677.1275; *m*/*z* 559.1229; 517.1140; and *m*/*z* 407.0762).

The pentamer, composed of five flavanol units (i.e., epicatechin or catechin), exhibits the same fragmentation pattern as the tetramer, identified by NMR [12] and fragmented in an earlier work [16]. The fragments resulting from RDA fission, with a loss of −152.0473 Da (-C_8_H_8_O_3_), are the same as those obtained for the tetramer (i.e., including fragments at *m*/*z* 1001.1868, at *m*/*z* 713.1370, and at *m*/*z* 425.0795 and one specifically for the pentamer at *m*/*z* 1289.2511). The fragments obtained by RDA fission are also the same as those found for the tetramer (-C_6_H_6_O_3_, fragment at *m*/*z* 739.1617 and at *m*/*z* 451.0966). Fragments from water loss (−18.0106 Da) were also observed (*m*/*z* 1424.2886, *m*/*z* 1271.2427, *m*/*z* 1253.2401, *m*/*z* 1137.2086, *m*/*z* 1119.1985, *m*/*z* 1010.1850, *m*/*z* 983.1806, *m*/*z* 847.1700, *m*/*z* 695.1249, *m*/*z* 677.1160, and *m*/*z* 559.1134). Furthermore, five ions with a mass difference of 288 Da (-C_15_H_12_O_6_) *m*/*z* 1441.2983, *m*/*z* 1153.2374, *m*/*z* 865.1810, *m*/*z* 577.1251, and *m*/*z* 289.0674)) were also found. All of these ions resulted from successive QM fissions from the crown pentamer (*m*/*z* 1153.2551). As for the other crown procyanidins, there was no observed loss of −290.0790 Da, which further supports the conclusion that the molecule is cyclic.

### 3.4. Quantification of Crown Procyanidines in Red and White Wines

Using the developed and validated UPLC-Q-TOF method described above, the following crown tannins were detected and quantified in different wines based on the molecular ion mass obtained in positive mode: tetramer (*m*/*z* 1153.2614), mono-gallo tetramer (*m*/*z* 1169.2563), and pentamer (*m*/*z* 1441.3248).

**Figure 2:**
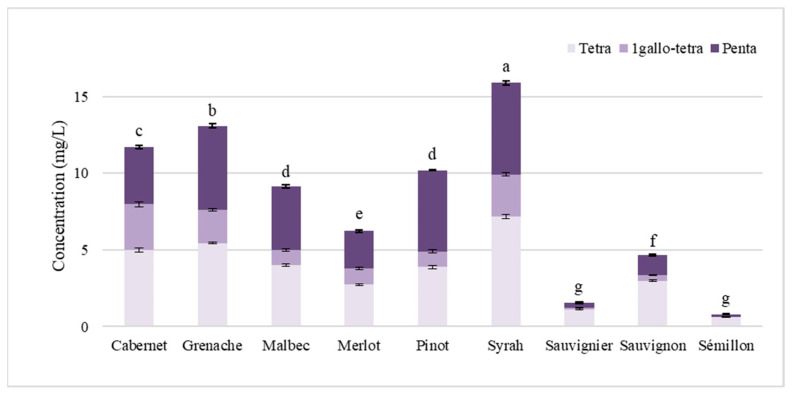
Concentrations of the main crown procyanidins. Significant differences, denoted by letters, were established from the total concentration of crown procyanidins.

Six red wines and three white wines were analysed by UHPLC-Q-TOF-MS, and the crown procyanidins were quantified as crown procyanidin tetramer equivalents. All results are reported in Figure 2. For the first time, crown tannins were quantified in various white wines. The crown procyanidin concentration ranged from 0.81 mg/L in Sémillon white wine to 15.88 mg/L for Syrah red wine. In all wines analysed, the crown procyanidin tetramer was found to be the major crown procyanidin. The concentrations in the studied white wines were between three and five times lower than those in red wines, which can be attributed to the differences in the maceration time.

## 4. Discussion

This study represents a significant advancement in the quantification of crown procyanidins, a unique sub-family of condensed tannins characterised by their macrocyclic structure. The development and validation of the UHPLC-Q-TOF-MS method provides a robust analytical approach without matrix effects, achieving high sensitivity (LOQ = 0.033 mg/L), accuracy (88.21–107.64%), and reproducibility (RSD < 20%), as recommended by international standards (OIV-MA-AS1-12). All validation parameters and the quantification calibration curve were established using the pure crown procyanidin tetramer, obtained as a white powder through a two-step purification process. This new purification strategy represents the optimisation of a previously reported three-step method [12]. Additionally, Bordeaux Clairet wine was selected as the starting wine matrix, rather than a conventional red wine with a more complex composition and higher phenolic content. This choice resulted in a more efficient and streamlined purification process, leading to the crown procyanidin tetramer. This validated method will be very helpful to support further research on the role of crown procyanidins in wine quality, but also offers the opportunity to explore their influence on the sensory properties of wine, such as colour stability, bitterness, and astringency, as well as its application in other food and beverage matrices containing similar compounds.

The application of new, validated method in the quantification of crown procyanidins in different wine matrices reveals, for the first time, the presence of crown procyanidins in white wine, as well as their significantly higher abundance in red wines. The concentrations ranged from 0.81 mg/L in Sémillon white wine to 15.88 mg/L in Syrah red wine. This disparity can be attributed to differences in the winemaking processes [29]. Indeed, crown procyanidins are exclusively found in the skin of grape berries; during white wine production, the grapes are pressed before alcoholic fermentation, resulting in minimal contact between the juice and the berry skins. This short contact time significantly limits the extraction of phenolic compounds from the skin, including crown procyanidins. In contrast, red wine production involves an extended maceration period throughout fermentation and often beyond, allowing for the more thorough extraction of the phenolics from the grape skins.

Even if the concentrations of the crown mono-gallo-proanthocyanidin tetramer and the crown pentamer were simply expressed as equivalents of the crown procyanidin tetramer—acknowledging that this might introduce minor discrepancies due to potential differences in ionisation and response factors—this validated method represents a significant step forward in the study of these new sub-families of condensed tannins.

## 5. Conclusions

This study presents the development and validation of a robust UHPLC-Q-TOF-MS method for the accurate quantification of the crown procyanidins in red and white wines, marking an advancement in the accurate quantification of this new sub-family of condensed tannins. This method effectively mitigates matrix effects and demonstrates high precision and reliability across wine types. Additionally, the validation parameters, including the linearity, accuracy, repeatability, and reproducibility, meet international standards, confirming that this method provides precise and reliable measurements. This advancement not only supports further research on the role of crown procyanidins in wine quality but also offers the opportunity to explore their influence on the organoleptic properties of wine, such as colour stability, bitterness, and astringency, as well as its application in other food and beverage matrices containing similar compounds.

Using this method, crown procyanidins were quantified for the first time in various white wines. Moreover, it was confirmed that crown procyanidins, particularly tetramers, are present at significantly higher concentrations in red wines compared to white wines, which aligns with the differences in the maceration processes. The extended maceration in red wine production allows for the enhanced extraction of these compounds from grape skins, whereas the brief contact during white wine production limits their presence.

Ultimately, this validated method offers a critical tool to advance our understanding of the polyphenolic profiles in winemaking and contributes to the broader field of wine composition research. Indeed, future research could expand this methodology to other food and beverage matrices where crown procyanidins might be present, yielding further knowledge regarding their organoleptic properties.

## Figures and Tables

**Table 1:** Validation parameters (calibration curve parameters, LOD and LOQ, repeatability, reproducibility, and accuracy) of the UHPLC-Q-TOF method.

		Red	White
Matrix effect (Z-score)		1.779	1.894
Calibration curves	Concentration range	0–1 mg/L	
	R^2^ (linear)	0.9919	
	Slope (x1000)	120,088	
	Lack of fit (Ftab/Fcalc)	2.57/1.05	
Detection and quantification limits	LOD	0.011 mg/L	
	LOQ	0.033 mg/L	
Accuracy and intraday precision (repeatability)	Spiking 0.1 mg/L		
	RSD (%)	11.03	8.77
	Recovery (%)	91.48	88.21
	Spiking 0.25 mg/L		
	RSD (%)	5.74	5.50
	Recovery (%)	98.42	98.10
	Spiking 0.5 mg/L		
	RSD (%)	7.37	1.99
	Recovery (%)	100.97	107.64
Intraday precision (reproducibility)	Spiking 0.05 mg/L		
	RSD (%)	14.49	14.62
	Spiking 0.1 mg/L		
	RSD (%)	19.05	13.63
	Spiking 0.5 mg/L		
	RSD (%)	6.80	2.51

**Table 2:** List of main crown procyanidins fragmented.

Compound	Retention Time (min)	Molecular Ion Formula	*m*/*z* ([M + H]^+^)	MS/MS Fragments
Tetramer [16]	2.8	C_60_H_49_O_24_	1153.2614	1135.2838, 1121.5960, 1001.2113, 983.1822, 1822, 865.2096, 739.1755, 713.1552, 695.1287, 677.1010, 577.1309, 559.1110, 517.5718, 451.1119, 433.0919, 425.0900, 289.0702
Mono-gallo tetramer	2.3	C_60_H_49_O_25_	1169.2551	1151.2519, 1017.2048, 999.1925, 981.1836, 881.1918, 865.1924, 847.1735, 755.1612, 739.1755, 729.1456, 713.1552, 695.1406, 677.1275, 593.1310, 577.1309, 559.1229, 467.0955, 451.1021, 441.0851, 425.0873, 407.0762, 305.0633
Pentamer	2.9	C_75_H_61_O_30_	1441.3248	1423.2865, 1289.2548, 1217.2427, 1253.2401, 1153.2374, 1137.2086, 1119.1985, 1101.1850, 1001.2016, 983.1806, 865.1810, 847.1700, 739.1617, 713.1345, 695.149, 677.1160, 577.1251, 559.1134, 451.0966, 425.0820, 289.0674

## Data Availability

The data that support the findings of this study are available on request from the corresponding author, M.J.

## Supplementary Materials

The following supporting information can be downloaded at: https://www.mdpi.com/article/10.3390/mps8010007/s1, Figure S1. Linearity curve of crown tetramer procyanidin; Table S1. Area of crown tetramer procyanidin in different matrices; Table S2. Data on matrix effect; Table S3. Values of the three ranges for the linearity curve; Table S4. Data on lack of fit; Table S5. Data on intraday repeatability and accuracy; Table S6. Data on intraday reproducibility; Table S7. Data on crown procyanidin quantification in each wine; Supplementary Information SI S1.
