# Supplementary material for: Development and Validation of a High-Throughput Quantification Method of Crown Procyanidins in Different Wines by UHPLC-Q-TOF
Source: Methods Protoc. 2025 Jan 11;8(1):7. doi: 10.3390/mps8010007 (PMC11755646; doi:10.3390/mps8010007)
Supplement: Supplementary file 1 [file mps-08-00007-s001.zip › mps-3354759-supplementary.pdf]

Supplementary information for article "Development and validation of a high-throughput quantification method of crown procyanidins in different wines by UHPLC–QTof"

Method validation

Table S1. Area of crown tetramer procyanidin in different matrices

	Red wine	Red wine doped (0.1mg/L)	Average red wine - red wine doped (0.1mg/L)
1	51045	63680	13589
2	49122	65310	15219
3	50105	64905	14814
moyenne	50091		

	White wine	white wine doped (0.1mg/L)	Average white wine - white wine doped (0.1mg/L)
1	7579	22851	14604
2	8148	24271	16024
3	9013	23429	15182
moyenne	8247		

	milliQ water doped (0.1mg/L)
1	15654
2	12527
3	14090

	Red wine	Red wine doped (0.1 mg/L)	Red wine doped (0.1 mg/L) - Red wine
Replicate 1	51045	63680	13589
Replicate 2	49122	65310	15219
Replicate 3	50105	64905	14814
Average	50091		

	White wine	white wine doped (0.1 mg/L)	White wine doped (0.1 mg/L) - White wine
Replicate 1	7579	22851	14604
Replicate 2	8148	24271	16024
Replicate 3	9013	23429	15182
Average	8247		

	milliQ water doped (0.1mg/L)
Replicate 1	15654
Replicate 2	12527
Replicate 3	140901

Table S2. Data of matrix effect

	Homocedasticity		Matrix effect			
	F calculed	F table	S^2_{pooled}	S_{Diff}	M_{Diff}	Z-score
Water VS Red wine	3.40	19	1582395	1027	1827	1.779
Water VS White wine	4.79	19	1477220	992	1880	1.894

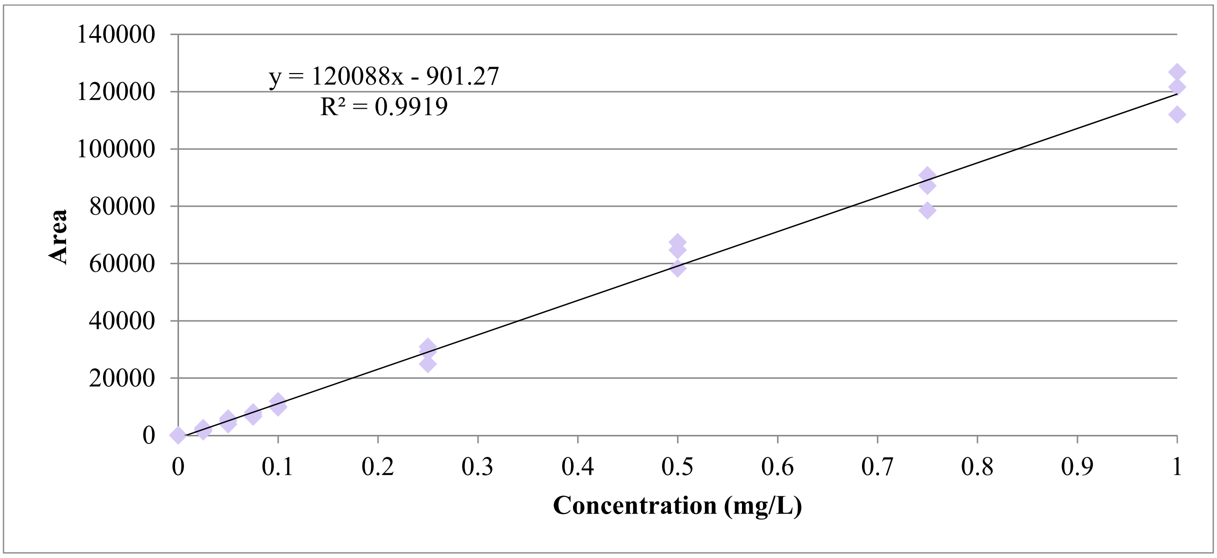

Figure S1 Linearity curve of crown tetramer procyanidin

Table S3. Values of the three range for linearity curve

Concentration (mg/L)	0	0.025	0.05	0.075	0.1	0.25	0.5	0.75	1
Range 1	0	2525	5884	7995	11916	29159	67436	90800	126799
Range 2	0	2000	5014	7239	10109	30928	64685	87096	121598
Range 3	0	1416	3879	6537	9717	24902	58288	78483	111988

Table S4. Data of Lack of fit

Source of variation	Sum-of-Squares	Degrees of freedom	Mean square	$F_{calculed}$	F Test de Fisher table (a=0.05; k-2. n-k)	Results
Residue	369945957	25	14797838			*Linear regression* $F_{calculed} < F_{table}$
Non-linear	107457069	7	15351010	1.0527	2.577	
Pure error	262488888	18	14582716			

Table S5. Data of intraday repeatability and accuracy

calculed value			average	standard deviation	RSD (%)	accuracy (%)
Replicate 1	Replicate 2	Replicate 3				

Red wine	Spiked 0.1mg/L	0.12	0.10	0.11	0.11	0.01	11.03	91.48
	Spiked 0.25mg/L	0.25	0.27	0.24	0.25	0.01	5.74	98.42
	Spiked 0.5mg/L	0.54	0.47	0.48	0.50	0.04	7.37	100.97
White wine	Spiked 0.1mg/L	0.12	0.10	0.11	0.11	0.01	8.77	88.21
	Spiked 0.25mg/L	0.27	0.24	0.25	0.25	0.01	5.50	98.10
	Spiked 0.5mg/L	0.46	0.47	0.46	0.46	0.01	1.99	107.64

	**Spiked**	**Replicate 1**	**Replicate 2**	**Replicate 3**	**Average**	**Standard deviation**	**RSD (%)**	**Accuracy (%)**
**Red wine**	0.1 mg/L	0.12	0.10	0.11	0.11	0.01	11.03	91.48
	0.25 mg/L	0.25	0.27	0.24	0.25	0.01	5.74	98.42
	0.5 mg/L	0.54	0.47	0.48	0.50	0.04	7.37	100.97
**White wine**	0.1 mg/L	0.12	0.10	0.11	0.11	0.01	8.77	88.21
	0.25 mg/L	0.27	0.24	0.25	0.25	0.01	5.50	98.10
	0.5 mg/L	0.46	0.47	0.46	0.46	0.01	1.99	107.64

Table S6. Data of intraday reproducibility

	Spiked	Day 1	Day 2	Day 3	Day 4	Day 5	Average	Standard deviation	RSD (%)
Red wine	0.05 mg/L	0.05	0.05	0.05	0.06	0.04	0.05	0.01	14.49
	0.1 mg/L	0.11	0.10	0.08	0.11	0.07	0.10	0.02	19.05
	0.5 mg/L	0.50	0.50	0.42	0.48	0.46	0.47	0.03	6.80
White wine	0.05 mg/L	0.06	0.06	0.06	0.05	0.08	0.06	0.01	14.62
	0.1 mg/L	0.14	0.11	0.11	0.10	0.10	0.11	0.02	13.63
	0.5 mg/L	0.46	0.46	0.44	0.44	0.45	0.45	0.01	2.51

Table S7. Data of different crown procyanidins quantification for each studied wine

		Concentration (mg/L)				
Wine	Replicate	Tetramer	Tetramer 1G	Pentamer	Total crown procyanidins	Average
Cabernet	1	4.86	3.17	3.79	11.82	11.70
	2	4.92	2.92	3.78	11.62	
	3	5.13	2.90	3.63	11.65	
Grenache	1	5.50	2.24	5.62	13.36	13.09

	2	5.40	2.16	5.32	12.88	
	3	5.46	2.04	5.53	13.03	
Malbec	1	3.69	0.79	4.06	8.54	9.14
	2	3.86	0.88	3.81	8.55	
	3	4.51	1.20	4.64	10.34	
Merlot	1	2.65	1.06	2.36	6.07	6.22
	2	2.78	1.11	2.54	6.43	
	3	2.76	0.95	2.44	6.16	
Pinot	1	3.77	0.97	5.30	10.04	10.18
	2	3.86	0.96	5.30	10.12	
	3	4.01	1.13	5.26	10.40	
Syrah	1	7.08	2.67	6.01	15.75	15.88
	2	7.14	2.84	5.80	15.78	
	3	7.33	2.70	6.08	16.11	
Sauvignier	1	1.17	0.10	0.32	1.59	1.55
	2	1.16	0.10	0.33	1.58	
	3	1.09	0.12	0.27	1.48	
Sauvignon	1	2.94	0.40	1.29	4.63	4.66
	2	2.99	0.37	1.25	4.61	
	3	3.06	0.36	1.33	4.75	
Sémillon	1	0.61	0.00	0.14	0.75	0.81
	2	0.67	0.00	0.19	0.86	
	3	0.64	0.00	0.19	0.83	

Supplementary information SI S1

```
Analysis of Variance Table

Response: DataValid$Crown
               Df  Sum Sq Mean Sq F value    Pr(>F)    
DataValid$Vins  8 1579.11 197.389  606.64 < 2.2e-16 ***
Residuals      18    5.86   0.325                      
---
Signif. codes:  0 ‘***’ 0.001 ‘**’ 0.01 ‘*’ 0.05 ‘.’ 0.1 ‘ ’ 1
$statistics
    MSerror Df     Mean       CV      MSD
  0.3253815 18 12.79222 4.459133 1.631916

$parameters
   test         name.t ntr StudentizedRange alpha
  Tukey DataValid$Vins   9         4.955209  0.05

$means
           DataValid$Crown        std r        se   Min   Max    Q25   Q50    Q75
Cabernet         18.373333 0.15502688 3 0.3293334 18.26 18.55 18.285 18.31 18.430
Grenache         20.486667 0.36774085 3 0.3293334 20.17 20.89 20.285 20.40 20.645
Malbec           14.500000 1.57619796 3 0.3293334 13.58 16.32 13.590 13.60 14.960
Merlot           10.050000 0.28583212 3 0.3293334  9.82 10.37  9.890  9.96 10.165
Pinot            16.046667 0.28746014 3 0.3293334 15.82 16.37 15.885 15.95 16.160
Sauvignier        2.406667 0.09237604 3 0.3293334  2.30  2.46  2.380  2.46  2.460
Sauvignon         7.246667 0.11718931 3 0.3293334  7.16  7.38  7.180  7.20  7.290
Semillon          1.256667 0.08736895 3 0.3293334  1.16  1.33  1.220  1.28  1.305
Syrah            24.763333 0.30088758 3 0.3293334 24.57 25.11 24.590 24.61 24.860

$comparison
NULL

$groups
           DataValid$Crown groups
Syrah            24.763333      a
Grenache         20.486667      b
Cabernet         18.373333      c
Pinot            16.046667      d
Malbec           14.500000      d
Merlot           10.050000      e
Sauvignon         7.246667      f
Sauvignier        2.406667      g
Semillon          1.256667      g

attr(,"class")
[1] "group"
```
